# Racial disparities in pain and total knee arthroplasty across knee osteoarthritis phenotypes

**DOI:** 10.3389/fragi.2026.1819274

**Published:** 2026-05-20

**Authors:** Ahmad Alkhatatbeh, Tariq Alkhatatbeh, Jiechen Chen, Hongjiang Chen, Jiankun Xu, Jun Hu

**Affiliations:** 1 Department of Orthopedics Surgery, The First Affiliated Hospital of Shantou University Medical College, Shantou, Guangdong, China; 2 Department of Joint Surgery, Center for Orthopedic Surgery, The Third Affiliated Hospital of Southern Medical University (Academy of Orthopedics, Guangdong Province), Guangzhou, Guangdong, China; 3 Orthopedic Hospital of Guangdong Province, The Third Affiliated Hospital of Southern Medical University, Guangzhou, Guangdong, China; 4 Guangdong Provincial Key Laboratory of Bone and Joint Degeneration Diseases, The Third Affiliated Hospital of Southern Medical University, Guangzhou, Guangdong, China; 5 Department of Sports Orthopaedics, TUM University Hospital, Technical University of Munich, Munich, Bavaria, Germany; 6 Musculoskeletal Research Laboratory, Centre for Musculoskeletal Degeneration & Regeneration, Department of Orthopaedics & Traumatology, Faculty of Medicine, The Chinese University of Hong Kong, Hong Kong SAR, China

**Keywords:** clinical phenotypes, electronic health records, health equity, knee osteoarthritis, machine learning, unsupervised clustering

## Abstract

**Introduction:**

Knee osteoarthritis is heterogeneous in radiographic severity, pain, and multimorbidity, and racial disparities in pain and knee replacement are documented. We derived knee osteoarthritis phenotypes from electronic health record and radiograph data and assessed their associations with race and incident total knee arthroplasty.

**Methods:**

We conducted a retrospective cohort study in the Emory Knee Radiograph dataset of adults aged ≥40 years with knee osteoarthritis (n = 33,553). Among participants with pain scores within ±90 days of the index radiograph (n = 20,030), we applied k-means clustering to features including age, radiographic severity, pain measures, and comorbidity indicators to define phenotypes. Among participants without pre-index strict total knee arthroplasty, we used Cox proportional hazards models to evaluate time to incident post-index strict total knee arthroplasty, with follow-up censored at last observed contact and capped at the procedure-code ascertainment horizon.

**Results:**

Clustering identified three phenotypes: structural/metabolic disease characterized by radiographic severity and multimorbidity; younger trauma-associated mild disease; and pain-dominant disease with pain disproportionate to radiographic severity. Black individuals were overrepresented in the pain-dominant phenotype and underrepresented in the structural/metabolic phenotype, whereas White individuals showed the opposite pattern. In time-to-event models, higher radiographic severity and male sex were associated with faster time to total knee arthroplasty, whereas the younger trauma-associated and pain-dominant phenotypes were associated with lower hazard of total knee arthroplasty than the structural/metabolic phenotype. These findings were preserved in known-race-only and categorical Kellgren–Lawrence grade sensitivity analyses.

**Discussion:**

Routinely available electronic health record and radiographic data can identify clinically meaningful knee osteoarthritis phenotypes that differ by race and are independently associated with subsequent total knee arthroplasty. These findings support further validation of phenotype-aware approaches to studying disparities and guiding osteoarthritis care.

## 1 Introduction

Knee osteoarthritis (KOA) is a leading cause of pain and disability, particularly among older adults and people with obesity (Steinmetz et al., 2023; Wang et al., 2024). The global burden of KOA attributable to high body mass index continues to rise (Miao et al., 2025). In the absence of disease-modifying therapies, care centers on symptom control and joint replacement. KOA is increasingly viewed as a heterogeneous syndrome with substantial variation in structural damage, pain, comorbidities, and treatment response (Dório and Deveza, 2022).

Discordance between radiographic severity and knee pain illustrates this heterogeneity: some individuals with clear radiographic KOA report little or no pain, whereas others have severe pain despite mild structural change (Dório and Deveza, 2022; Bedson and Croft, 2008; Neogi et al., 2009). This pain-structure mismatch affects decisions about total knee arthroplasty (TKA) and has motivated efforts to define KOA phenotypes that integrate symptoms and structural features (Dório and Deveza, 2022; Kittelson et al., 2016; Carlesso et al., 2022; Dell’Isola et al., 2016). Prior phenotyping studies have proposed mechanical, metabolic, and pain-dominant subgroups but often rely on small, selective cohorts with limited racial diversity and multimorbidity (Kittelson et al., 2016; Carlesso et al., 2022; Dell’Isola et al., 2016). Complementing phenotype-based approaches, prognostic models built from routinely available clinical variables can estimate long-term pain outcomes, including the probability of pain resilience, and thereby support risk stratification in KOA (Alkhatatbeh A. et al., 2026).

At the same time, multimorbidity is central to KOA trajectories and outcomes (Lentz et al., 2021). Large epidemiologic studies show osteoarthritis is accompanied by comorbidity clusters (e.g., cardiometabolic, musculoskeletal, and psychological) associated with differences in pain, function, and mortality (Neogi et al., 2009; Lentz et al., 2021; Pineda-Moncusí et al., 2023). However, most KOA phenotyping frameworks either ignore comorbidities altogether or treat them as simple covariates, and few have examined how comorbidities interact with radiographic severity and pain in routine care (Lentz et al., 2021; Pineda-Moncusí et al., 2023).

Electronic health records (EHR) systems and large open datasets now allow derivation of phenotypes that reflect real-world care by linking imaging, symptoms, and comorbidities at scale (Yang et al., 2023; Mullainathan and Obermeyer, 2022). The Emory Knee Radiograph (MRKR) dataset is one such resource, pairing de-identified knee radiographs from a large academic health system with longitudinal EHR data to enable joint analysis of radiographic severity, pain, multimorbidity, and arthroplasty (Price, 2025). Existing KOA phenotyping studies rarely use such imaging–EHR resources, often focus on either pain or comorbidities, and seldom evaluate how data-driven phenotypes relate to racial inequities in pain and TKA use (Kittelson et al., 2016; Dell’Isola et al., 2016; Lentz et al., 2021; Pineda-Moncusí et al., 2023). Our objective was to construct an EHR-derived KOA cohort from MRKR, derive phenotypes integrating radiographic severity, knee pain, and comorbidities, and test whether these phenotypes differ by race and add prognostic information about incident post-index TKA beyond age, sex, race, pain, and radiographic severity.

## 2 Materials and methods

### 2.1 Study design and data source

We conducted a retrospective cohort study using the publicly released Emory Knee Radiograph (MRKR) dataset, which links de-identified radiographic metadata, deep learning–derived Kellgren–Lawrence (KL) grades, numeric pain scores, diagnosis and procedure codes, and basic demographics for individuals who underwent knee radiography at a large US academic health system. The dataset creators report that the development of this retrospective dataset was approved by an Institutional Review Board. All data were fully de-identified before release. Our analysis used only de-identified data and therefore did not require additional institutional review board review; informed consent to participate was not required. All procedures were conducted in accordance with the Declaration of Helsinki and relevant guidelines and regulations.

### 2.2 Study population

We included adults aged 40 years or older with evidence of knee OA and at least one frontal knee radiograph in MRKR. For each examination, we computed the maximum KL grade across knees; radiographs with missing grades were excluded. For each individual, the earliest frontal radiograph with a nonmissing KL grade defined the index radiograph, and we recorded its date and age at examination.

### 2.3 Osteoarthritis definition and exclusions

Individuals were classified as having knee OA if they had radiographic OA at index (maximum KL grade ≥2) or at least one diagnosis code consistent with knee OA based on a predefined MRKR flag. These indicators were merged into the imaging cohort, and individuals meeting either criterion were retained. We excluded individuals whose index radiograph showed knee arthroplasty hardware and those younger than 40 years at the index date. The final analysis cohort comprised 33,553 individuals.

### 2.4 Demographic variables

Sex, race, and ethnicity were obtained from MRKR demographics files and merged with the index cohort using the anonymized individual identifier. Race and ethnicity were analyzed as recorded. The MRKR files available for this study do not document whether race was self-reported, registrar-entered, or otherwise collected. Because the dataset included an explicit Unknown race category, we additionally summarized characteristics of individuals with Unknown versus known race in the full cohort.

### 2.5 Pain measures

Numeric rating scale (0–10) pain scores and a knee-specific indicator were obtained from MRKR pain tables, which include the score, encounter date, and a binary knee-specific flag. We linked pain records to the index radiograph and restricted analyses to scores within 90 days before or after the index date. Among knee-specific records in this window, we defined three individual-level measures: median knee pain, the proportion of scores ≥7, and an indicator of any knee-specific pain data. Individuals with at least one knee-specific pain score comprised the pain subset for clustering (n = 20,030); those without such scores remained in the overall cohort for descriptive and arthroplasty analyses only.

### 2.6 Comorbidities

Comorbidities were derived from ICD-9 and ICD-10 diagnosis codes recorded on or before the index date. Using precomputed MRKR flags, we created individual-level indicators for diabetes, hypertension, obesity, autoimmune disease, lower extremity trauma, nicotine use, and knee OA, coded as present if any qualifying pre-index diagnosis was recorded.

In addition, we defined chronic kidney disease and cardiovascular disease using standard ICD-9/10 code sets for chronic kidney disease and for ischemic heart disease or heart failure. Comorbidity prevalences refer to these pre-index baseline indicators.

### 2.7 Arthroplasty procedures and outcome definition

Strict total knee arthroplasty (TKA) procedures were identified in MRKR procedure tables using Current Procedural Terminology (CPT) codes 27447, 27486, and 27487. For each individual, we determined the earliest qualifying strict TKA date and classified it as pre-index (before index) or incident post-index (on or after index).

For time-to-event analyses, follow-up began at the index radiograph date and ended at the earliest of incident strict TKA or censoring. Censoring was defined as the last observed contact date across MRKR imaging, pain, diagnosis, and procedure tables, capped at the CPT ascertainment horizon. Follow-up time was calculated as days from index to event or censoring, with 1 day added to retain same-day events and same-day censoring. The primary outcome analysis used time to incident post-index strict TKA among individuals in the pain subset without pre-index strict TKA.

### 2.8 Clustering features and preprocessing

Clustering was restricted to the pain subset. For each individual in this subset, we constructed a feature vector comprising age at index, maximum KL grade, median knee pain score, the proportion of knee pain scores ≥7, and binary indicators for hypertension, obesity, diabetes, cardiovascular disease, chronic kidney disease, autoimmune disease, lower extremity trauma, and nicotine use. Individuals missing any of these features were excluded from clustering. In practice, no additional exclusions occurred after restricting to individuals with at least one knee-specific pain score within ±90 days of the index radiograph.

Continuous variables were standardized to mean 0 and standard deviation (SD) of 1; binary indicators were coded as 0 or 1. The resulting standardized feature matrix was used as input to clustering.

### 2.9 Unsupervised clustering, phenotype definition, and stability

We applied k-means clustering to the standardized feature matrix and evaluated solutions with 3–6 clusters, fitting each model with 20 initializations and a fixed random seed for reproducibility. For each solution, we examined within-cluster sum of squares (inertia), the silhouette coefficient, and the Calinski-Harabasz index. We selected K = 3 as the primary solution based on the elbow in the inertia plot, reasonable silhouette and Calinski-Harabasz values, and the clinical interpretability and stability of the resulting clusters. Cluster profiles were summarized and used to assign descriptive labels: phenotype 0 (structural/metabolic advanced OA), phenotype 1 (younger, trauma-associated mild OA), and phenotype 2 (pain-dominant OA). Indicator variables for phenotypes 1 and 2, with phenotype 0 as the reference, were used in subsequent models.

Cluster stability was assessed using 100 random 80% subsamples of the pain subset, which showed high agreement between refitted and original cluster assignments.

### 2.10 Pain-structure residuals

To quantify discordance between structural severity and pain, we modeled median knee pain as a function of maximum KL grade, age, sex, and race in the pain subset using linear regression. For each individual, we calculated a pain residual as observed minus predicted median pain. Positive residuals indicate higher-than-expected pain given structural severity and demographics, whereas negative residuals indicate lower-than-expected pain. Residuals were summarized by phenotype and visualized with boxplots.

### 2.11 Race and ethnicity distribution by phenotype

Race and ethnicity distributions were summarized within each phenotype using cross-tabulations of phenotype by race and, when available, ethnicity. We reported counts and within-phenotype percentages to describe differences in racial and ethnic composition across phenotypes.

### 2.12 Association between phenotypes and incident TKA

Among individuals in the pain subset without pre-index strict TKA, we used multivariable Cox proportional hazards regression to examine the association between phenotype membership and incident post-index strict TKA. The outcome was time from the index radiograph to incident strict TKA, with censoring at last observed contact capped at the CPT ascertainment horizon. Independent variables were maximum KL grade, median knee pain score, indicators for phenotypes 1 and 2 (phenotype 0 as reference), age at index, sex, and race. Continuous predictors were standardized, and hazard ratios for these variables are reported per SD increase. Phenotype coefficients represent the association between phenotype membership and the hazard of incident post-index TKA after adjustment for radiographic severity, pain, age, sex, and race. Proportional hazards diagnostics were assessed for the primary model. To complement relative hazard estimates, we also estimated unadjusted Kaplan–Meier cumulative incidence of strict TKA at 1 and 3 years within the primary Cox cohort.

### 2.13 Sensitivity analyses

We conducted six sensitivity analyses. First, we repeated k-means clustering with 4- and 5-cluster solutions using the same standardized feature set to assess whether additional clusters yielded materially different or more clinically interpretable phenotypes. Second, we re-estimated the 3-cluster solution after excluding all individuals with any record of TKA to evaluate whether arthroplasty influenced cluster structure. Third, we compared individuals with and without a knee-specific pain score within ±90 days of the index radiograph to assess potential selection bias. Fourth, we repeated pain-feature construction and phenotype derivation using a narrower ±30-day pain window to assess robustness to the primary window choice. Fifth, we repeated the primary Cox model after excluding individuals with unknown race and after modeling maximum KL grade categorically rather than as a standardized continuous term. Sixth, we repeated the primary Cox model after excluding individuals censored on the index date to assess whether retaining same-day censoring observations via the +1-day follow-up adjustment influenced the results.

### 2.14 Statistics and reproducibility

We analyzed all eligible individuals meeting inclusion criteria; no *a priori* power calculation was performed because this was a retrospective study using an existing dataset. Continuous features were standardized (mean 0, SD 1) prior to clustering. Unsupervised phenotyping used k-means clustering, as detailed in Section 2.9. Associations with incident post-index strict total knee arthroplasty were evaluated using multivariable Cox proportional hazards regression with two-sided tests and α = 0.05, reporting hazard ratios with 95% confidence intervals and exact P values. Proportional hazards diagnostics were assessed for the primary model. Pain–structure residuals were estimated using linear regression adjusted for maximum Kellgren–Lawrence grade, age, sex, and race. We also summarized patient-level availability of repeated radiograph, pain, diagnosis, and procedure dates to contextualize the feasibility of future longitudinal phenotype analyses.

Cluster stability (reproducibility) was assessed using 100 random 80% subsamples and agreement between refit and original assignments (adjusted Rand index).

Analyses were conducted in Python (v3.11) using standard scientific libraries (e.g., numpy, pandas, scikit-learn, statsmodels), with full software/package versions recorded in the analysis environment file.

## 3 Results

### 3.1 Cohort characteristics

The cohort included 33,553 individuals (mean age, 61.2 [SD, 10.9] years; mean maximum Kellgren–Lawrence grade, 2.34 [SD, 1.10]). Baseline comorbidities were common: 42.3% had hypertension, 22.6% obesity, 14.6% diabetes, 14.8% cardiovascular disease, 6.0% chronic kidney disease, 6.0% autoimmune disease, 22.6% a history of lower extremity trauma, and 10.5% documented nicotine use (Table 1). A total of 20,030 individuals had at least 1 knee-specific pain score within ±90 days of the index radiograph and comprised the clustering subset. The remaining 13,523 individuals were excluded from clustering because they lacked a knee-specific pain score within this window. No additional exclusions occurred because of missing clustering features after the pain-window restriction. Comparisons of included and excluded individuals and clustering-feature missingness are provided in Supplementary Tables S1, S2, and the full cohort flow is shown in Supplementary Figure S1.

**TABLE 1:** Baseline characteristics of the Emory Knee Radiograph (MRKR) knee osteoarthritis cohort (N = 33,553).

Characteristic	Value
Continuous variables	
Age at index, years	61.2 ± 10.9
Median (IQR)	61 (53–69)
Maximum Kellgren–Lawrence grade	2.34 ± 1.10
Median (IQR)	2 (2–3)
Binary variables	Proportion
Hypertension (HTN)	42.3%
Obesity	22.6%
Diabetes	14.6%
Cardiovascular disease (CVD)	14.8%
Chronic kidney disease (CKD)	6.0%
Autoimmune disease	6.0%
Lower extremity trauma	22.6%
Nicotine use (ever)	10.5%
Comorbidities reflect pre-index diagnoses only	

Continuous variables are mean ± standard deviation (SD) or median (interquartile range); binary variables are proportions. Baseline comorbidities are defined from ICD codes recorded prior to the index radiograph.

### 3.2 Phenotypes derived from imaging, pain, and comorbidities

**FIGURE 1:**
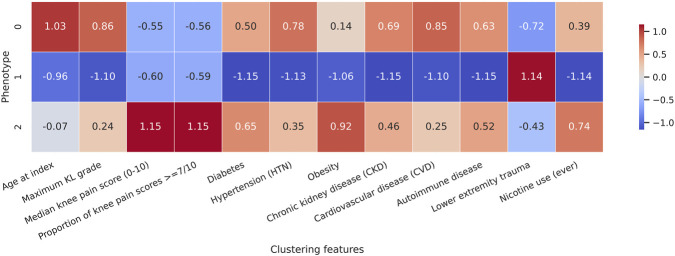
Standardized feature profiles of data-driven knee osteoarthritis phenotypes. Heatmap showing mean standardized values (z-scores) of age, radiographic severity, knee pain measures, and comorbidities for each phenotype. The structural/metabolic phenotype shows high radiographic severity and cardiometabolic burden; the younger, trauma-associated mild OA phenotype shows lower radiographic severity and high lower extremity trauma; and the pain-dominant OA phenotype shows moderate radiographic severity but markedly elevated pain. Abbreviations: HTN: hypertension; CVD: cardiovascular disease; CKD: chronic kidney disease; LE: lower extremity.

**FIGURE 2:**
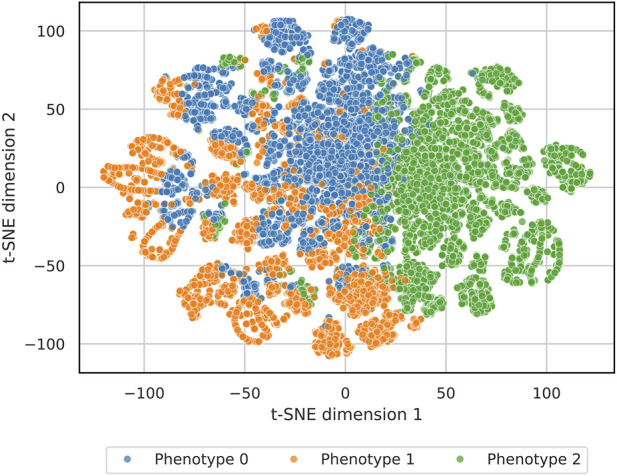
Two-dimensional embedding of patients colored by knee osteoarthritis phenotype. t-SNE plot of the standardized clustering feature space for patients with knee pain data, with points colored by assigned phenotype. The plot illustrates the separation of the structural/metabolic, younger trauma-associated, and pain-dominant phenotypes in the multivariate feature space.

Among individuals with knee-specific pain data, k-means clustering (K = 3) of standardized imaging severity, pain measures, and comorbidities identified three phenotypes with distinct profiles (Table 2; Figures 1, 2).

**TABLE 2:** Data-driven knee OA phenotypes: characteristics and race distribution.

Characteristic	Phenotype 0 N = 6,658	Phenotype 1 N = 6,134	Phenotype 2 N = 7,238
Demographics and structural			
Age at index, years	69.9 ± 8.0	54.2 ± 7.9	61.2 ± 10.7
Maximum KL grade	2.87 ± 0.85	1.69 ± 1.00	2.50 ± 1.07
Pain measures			
Median knee pain (0–10)	3.98 ± 1.70	3.86 ± 1.71	8.07 ± 1.09
High knee pain (≥7/10), %	7.9%	6.5%	92.5%
Pain residual (pain-structure discordance)	−1.33 ± 1.76	−1.36 ± 1.76	2.37 ± 1.21
Outcome			
Ever total knee arthroplasty (TKA), %	85.2%	22.6%	54.5%
Comorbidities			
Hypertension (HTN), %	66.6%	32.2%	58.8%
Obesity, %	33.2%	23.0%	40.0%
Diabetes, %	23.1%	9.7%	24.4%
Cardiovascular disease (CVD), %	25.5%	8.6%	20.3%
Chronic kidney disease (CKD), %	11.7%	3.6%	10.7%
Autoimmune disease, %	10.0%	4.5%	9.7%
Lower extremity trauma, %	24.8%	46.8%	28.2%
Nicotine use (ever), %	18.4%	10.2%	20.2%
Race and ethnicity			
Black, n (%)	1,934 (29.0%)	1,891 (30.8%)	3,908 (54.0%)
White, n (%)	3,923 (58.9%)	3,051 (49.7%)	2,176 (30.1%)
Asian, n (%)	234 (3.5%)	257 (4.2%)	269 (3.7%)
Other*, n (%)	59 (0.9%)	70 (1.1%)	80 (1.1%)
Unknown, n (%)	508 (7.6%)	865 (14.1%)	805 (11.1%)

* Other = American Indian or Alaska Native, multiple race, and Native Hawaiian or other Pacific Islander. Percentages sum to 100% within each phenotype, allowing for rounding. Values are mean ± SD, for continuous variables and proportions for binary variables. “structural/metabolic advanced OA” (phenotype 0), “younger, trauma-associated mild OA” (phenotype 1), and “pain-dominant OA” (phenotype 2) are labeled based on their dominant features.

Pain residuals were calculated from a linear regression model of median knee pain on maximum Kellgren–Lawrence grade, age, sex, and race. Negative values indicate lower-than-expected pain, and positive values indicate higher-than-expected pain. The overall mean residual in the fitted sample was effectively zero (4.8 × 10^-16), as expected for an ordinary least-squares model with an intercept.

Abbreviations: OA, osteoarthritis; KL grade, Kellgren–Lawrence grade; SD, standard deviation.

Phenotype 0 (“structural/metabolic advanced OA”; n = 6,658) included the oldest individuals (mean age, 69.9 years) and the greatest structural severity (mean maximum Kellgren–Lawrence grade, 2.87). Median knee pain was 3.98 of 10, and 7.9% of pain scores were ≥7; 85.2% had ever undergone total knee arthroplasty (TKA). Cardiometabolic comorbidities were frequent (hypertension, 66.6%; obesity, 33.2%; diabetes, 23.1%; cardiovascular disease, 25.5%; chronic kidney disease, 11.7%), and the mean pain residual was −1.33.

Phenotype 1 (“younger, trauma-associated mild OA”; n = 6,134) comprised younger individuals (mean age, 54.2 years) with the lowest structural severity (mean maximum Kellgren–Lawrence grade, 1.69). Median knee pain was similar to phenotype 0 (3.86 of 10), and 6.5% of scores were ≥7. Metabolic comorbidities were less frequent (hypertension, 32.2%; obesity, 23.0%; diabetes, 9.7%; cardiovascular disease, 8.6%), whereas lower extremity trauma was common (46.8%). The prevalence of ever having undergone TKA was 22.6%, and the mean pain residual was −1.36.

Phenotype 2 (“pain-dominant OA”; n = 7,238) had intermediate age (mean, 61.2 years) and structural severity (mean maximum Kellgren–Lawrence grade, 2.50) but very high pain (median, 8.07 of 10; 92.5% of scores ≥7). Hypertension (58.8%), obesity (40.0%), diabetes (24.4%), and cardiovascular disease (20.3%) were of intermediate frequency; 54.5% had ever undergone TKA. The mean pain residual was +2.37, indicating substantially higher pain than expected given radiographic severity and demographics. A two-dimensional embedding of the clustering feature space showed good separation and limited overlap among the three phenotypes (Figure 2).

### 3.3 Race and ethnicity distribution across phenotypes

Race distribution differed across phenotypes (Table 2). In the pain-dominant OA phenotype, 54% of individuals were Black, 30% White, 4% Asian, and 11% had unknown race. The structural/metabolic advanced OA phenotype was majority White (59% White, 29% Black, 4% Asian, 8% unknown), and the younger, trauma-associated phenotype had an intermediate pattern (50% White, 31% Black, 4% Asian, 14% unknown). Thus, Black individuals were relatively overrepresented in the pain-dominant OA phenotype and underrepresented in the structural/metabolic advanced OA phenotype, whereas White individuals showed the opposite pattern.

In a separate full-cohort descriptive comparison, individuals with Unknown race were younger than those with known race (mean age, 58.8 vs. 61.5 years) and had markedly shorter observed follow-up to strict TKA event or censoring (615.6 vs. 1394.9 days). They also had lower incident post-index strict TKA (6.8% vs. 17.2%) and lower recorded cardiometabolic comorbidity burden, including lower prevalences of hypertension (18.5% vs. 53.7%), obesity (11.9% vs. 30.8%), and diabetes (6.9% vs. 18.7%) (Supplementary Table S3).

### 3.4 Pain-structure residuals

**FIGURE 3:**
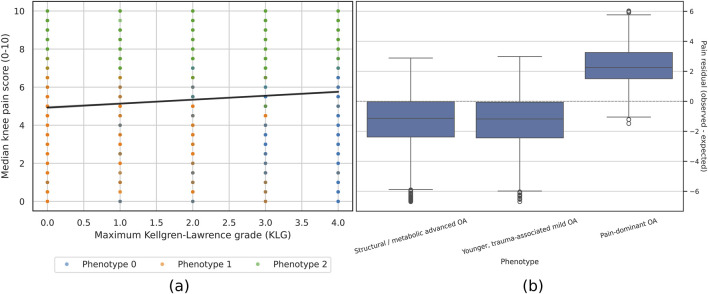
Pain-structure relationship and pain residuals by knee osteoarthritis phenotype. **(a)** Scatter plot of median knee pain score versus maximum Kellgren–Lawrence grade, with points colored by phenotype. The fitted regression line shows the average pain-structure relationship. **(b)** Boxplots of patient-level residuals from a linear model of median knee pain on maximum Kellgren–Lawrence grade, age, sex, and race, stratified by phenotype. Positive values indicate higher-than-expected pain, and negative values indicate lower-than-expected pain.

Figure 3 summarizes the relationship between radiographic severity and median knee pain and the distribution of pain-structure residuals by phenotype. Mean residuals were −1.33 for phenotype 0, −1.36 for phenotype 1, and +2.37 for phenotype 2, indicating lower-than-expected pain in the structural/metabolic and younger, trauma-associated phenotypes and substantially higher-than-expected pain in the pain-dominant OA phenotype.

### 3.5 Association of phenotypes with total knee arthroplasty

In multivariable Cox proportional hazards regression for incident post-index strict TKA among individuals in the pain subset without pre-index strict TKA (Table 3), higher maximum Kellgren–Lawrence grade was strongly associated with incident TKA (HR 2.00; 95% CI, 1.92–2.09 per 1-SD increase; P < 0.001). Median knee pain showed a weaker positive association (HR 1.05; 95% CI, 0.99–1.11; P = 0.083).

**TABLE 3:** Multivariable Cox proportional hazards model for incident post-index strict total knee arthroplasty.

Variable	Hazard ratio (95% CI)	P value
Age at index (per SD)	0.97 (0.94–1.01)	0.204
Maximum KL grade (per SD)	2.00 (1.92–2.09)	<0.001
Median knee pain (per SD)	1.05 (0.99–1.11)	0.083
Phenotype (ref: structural/metabolic advanced OA; phenotype 0)		
Younger, trauma-associated mild OA (phenotype 1)	0.47 (0.42–0.54)	<0.001
Pain-dominant OA (phenotype 2)	0.73 (0.65–0.83)	<0.001
Sex		
Male (ref: Female)	1.14 (1.07–1.23)	<0.001
Race (ref: Black)		
American Indian or Alaska Native	1.55 (0.91–2.62)	0.104
Asian	1.48 (1.24–1.76)	<0.001
White	1.50 (1.39–1.62)	<0.001
Multiple	1.70 (1.09–2.65)	0.018
Native Hawaiian or other Pacific Islander	1.42 (0.63–3.16)	0.396
Unknown	0.98 (0.84–1.15)	0.839

Hazard ratios are from a multivariable Cox proportional hazards regression model for incident post-index strict TKA, adjusted for age at index, maximum KL, grade, median knee pain, phenotype, sex, and race. Reference categories are indicated in parentheses.

Abbreviations: CI, confidence interval; KL grade, Kellgren–Lawrence grade; SD, standard deviation; TKA, total knee arthroplasty; OA, osteoarthritis.

Using the structural/metabolic advanced OA phenotype as the reference, hazard of incident TKA was lower in the younger, trauma-associated mild OA phenotype (HR 0.47; 95% CI, 0.42–0.54; P < 0.001) and in the pain-dominant OA phenotype (HR 0.73; 95% CI, 0.65–0.83; P < 0.001). Male sex was associated with slightly higher hazard of TKA (HR 1.14; 95% CI, 1.07–1.23; P < 0.001). Compared with Black individuals, hazard of TKA was higher in Asian (HR 1.48; 95% CI, 1.24–1.76; P < 0.001) and White (HR 1.50; 95% CI, 1.39–1.62; P < 0.001) individuals, whereas the Unknown race category was not significantly associated with incident TKA (HR 0.98; 95% CI, 0.84–1.15; P = 0.839); estimates for smaller race groups were less precise. Proportional hazards diagnostics for the primary model are summarized in Supplementary Table S4.

Phenotype-stratified absolute-risk summaries were consistent with the adjusted hazard ratios (Supplementary Table S5). In the primary Cox cohort, Kaplan–Meier estimated cumulative strict-TKA incidence at 1 year was 20.1% for the structural/metabolic phenotype, 4.3% for the younger trauma-associated phenotype, and 12.6% for the pain-dominant phenotype; corresponding 3-year cumulative incidences were 27.8%, 6.9%, and 18.3%, respectively.

### 3.6 Alternative cluster numbers

Clustering with K = 4 and K = 5 yielded only modest changes in silhouette coefficients relative to K = 3, with values approximately 0.176–0.181, and the Calinski-Harabasz index did not clearly improve, with values of approximately 5,355 for K = 4 and 4,869 for K = 5. Additional clusters mainly split existing groups without revealing qualitatively new patterns, supporting K = 3 as the primary solution. In 100 random 80% subsamples, the adjusted Rand index comparing refitted with original K = 3 labels had a mean of 0.99 (range, 0.98–1.00), indicating high robustness of the three-phenotype solution.

### 3.7 Clustering among individuals without TKA

In a sensitivity analysis restricted to individuals who had never undergone TKA, K = 3 clustering produced qualitatively similar groups: an older, structurally advanced phenotype (maximum Kellgren–Lawrence grade ≈2.65; median pain ≈3.9), a pain-dominant OA phenotype (maximum Kellgren–Lawrence grade ≈2.38; median pain ≈8.1), and a younger, mild OA, trauma-enriched phenotype (maximum Kellgren–Lawrence grade ≈1.68; median pain ≈3.9, high lower extremity trauma). These findings suggest that the structural/metabolic advanced OA, pain-dominant OA, and younger, trauma-associated mild OA phenotypes are not solely driven by prior arthroplasty.

### 3.8 Pain-window and model robustness

Using a narrower ±30-day pain window, 18,082 individuals had knee-specific pain data. Phenotype structure remained similar to the primary ±90-day solution, with overlap adjusted Rand index 0.808 and mapped label agreement 93.4%, supporting the ±90-day window as the primary analysis while indicating robustness to a narrower window (Supplementary Table S6). In additional Cox sensitivity analyses, the main phenotype findings were preserved after excluding individuals with unknown race and after modeling maximum KL grade categorically rather than continuously (Supplementary Tables S7, S8). In the known-race-only model, median knee pain became nominally significant, but phenotype estimates remained materially similar. Excluding 1,070 individuals censored on the index date did not materially change the primary Cox estimates (e.g., maximum KL grade HR, 2.01; phenotype 1 HR, 0.47; phenotype 2 HR, 0.73), supporting robustness of the +1-day follow-up handling used to retain same-day censoring observations (Supplementary Table S9).

## 4 Discussion

### 4.1 Principal findings

In this large, racially diverse cohort of more than 33,000 individuals with knee OA, unsupervised clustering of radiographic severity, knee pain, and comorbidities identified 3 stable and clinically interpretable phenotypes: structural/metabolic, younger trauma-associated, and pain-dominant. The pain-dominant phenotype had severe knee pain and large positive pain-structure residuals despite only moderate Kellgren–Lawrence grades, whereas the structural/metabolic phenotype combined older age, more advanced radiographic change, cardiometabolic multimorbidity, and the highest prevalence of total knee arthroplasty (TKA). Phenotype membership remained associated with time to incident post-index strict TKA in Cox models after adjustment for radiographic severity, pain, age, sex, and race, indicating incremental prognostic information beyond standard covariates.

### 4.2 Comparison with previous phenotyping work

Systematic reviews have proposed that knee OA comprises partially distinct structural, metabolic, mechanical overload, and chronic pain phenotypes but emphasize the small size, selective enrollment, and limited racial diversity of most prior cohorts (Dell’Isola et al., 2018; Carlesso and Neogi, 2020). Our structural/metabolic phenotype resembles “severe structural osteoarthritis” groups and multimorbid cardiometabolic clusters reported previously (Dório and Deveza, 2022; Dell’Isola and Steultjens, 2018; Lentz et al., 2021; Pineda-Moncusí et al., 2023), whereas the younger, trauma-associated phenotype is consistent with mechanically driven or post-traumatic patterns. This aligns with growing evidence that metabolic dysregulation and systemic metabolites contribute to OA progression (Liu et al., 2025). The pain-dominant phenotype parallels “central sensitization” or high-pain sensitivity phenotypes identified using quantitative sensory testing and psychological measures (Kittelson et al., 2016; Carlesso et al., 2022). Recovering analogous patterns from deep learning–derived radiographic measures and routine numeric rating scale pain scores suggests that clinically meaningful phenotypes can be derived from standard electronic health record (EHR) data without specialized instruments.

### 4.3 Race, pain patterns, and access to arthroplasty

Racial differences in phenotype distribution and TKA use may reflect disparities in care processes, but these associations should be interpreted cautiously given potential unmeasured structural confounding. Black individuals were overrepresented in the pain-dominant phenotype and underrepresented in the structural/metabolic advanced OA phenotype, whereas White individuals showed the reverse pattern. Despite the highest pain burden, the pain-dominant phenotype had substantially lower adjusted hazard of incident TKA than the structural/metabolic phenotype. This pattern is consistent with literature documenting racial inequities in arthroplasty utilization (Singh et al., 2014; Cavanaugh et al., 2019), as well as a systematic review and meta-analysis showing worse postoperative outcomes for Black compared with White individuals undergoing TKA (Balachandran et al., 2025). One possible explanation is that if clinical decision-making emphasizes radiographic “bone-on-bone” severity over patient-reported pain, phenotypes characterized by high pain but only moderate structural damage, which were more common among Black individuals in this cohort, may be relatively deprioritized for surgery. Importantly, the Unknown race category should not be interpreted as benign random missingness. In descriptive analyses, individuals with Unknown race had substantially shorter observed follow-up, lower incident strict TKA, and lower recorded comorbidity burden than those with known race, and the MRKR files available for this study did not document whether race was self-reported, registrar-entered, or otherwise collected. We therefore treated this category cautiously and confirmed that the main phenotype findings were preserved in known-race-only models. Although we could not account for structural racism, payer/insurance, education, income, neighborhood deprivation, geographic access to orthopedic care, patient preferences or trust in the medical system, or clinician-, facility-, and system-level factors, these results are consistent with differences in disease expression and care processes that may interact, but they do not identify the causal mechanisms behind those differences.

### 4.4 Clinical and research implications

These EHR-deployable phenotypes have practical implications. Phenotype-stratified survival summaries provide more actionable absolute-risk context: in the primary Cox cohort, 1-year cumulative strict-TKA incidence was 20.1% in the structural/metabolic phenotype, 4.3% in the younger trauma-associated phenotype, and 12.6% in the pain-dominant phenotype; corresponding 3-year incidences were 27.8%, 6.9%, and 18.3%, respectively. Because phenotype assignment in this study relied only on routinely available variables, these phenotypes may serve as a pragmatic stratification layer for dashboards or triage-support workflows, but they should not be used as standalone treatment algorithms without external validation. Automated identification of a pain-dominant phenotype could prompt early multimodal pain management and referral for centrally mediated pain, rather than repeated mechanically focused interventions with limited benefit. The younger trauma-associated phenotype may reflect post-traumatic pathways, including ligament degeneration, which has been associated with incident knee OA (Luo et al., 2024). Accordingly, early biomechanical and rehabilitation strategies are being explored in KOA, including pendulum-based approaches evaluated in feasibility trials (Huang et al., 2024), and targeting injury-related pathways is increasingly important given the substantial global burden of severe athletic knee trauma in young adults (Alkhatatbeh T. et al., 2026). For structural/metabolic disease, the combination of advanced radiographic severity, cardiometabolic burden, and frequent TKA highlights the need for perioperative optimization; when full-length lower limb radiography is used to support alignment assessment for arthroplasty planning, automated stitching methods can streamline radiographic preparation (Alkhatatbeh et al., 2022). At the health system level, phenotype-stratified dashboards could support equity monitoring by examining TKA access and outcomes jointly by race and phenotype. Research priorities include external validation in other health systems, assessment of generalizability across insurance and referral contexts, and longitudinal analyses of phenotype transitions and trajectories, extending prior work on pain phenotypes (Ye et al., 2024). Finally, combining phenotype assignment with prognostic nomograms for long-term pain outcomes (including pain resilience) may improve risk stratification in routine care (Alkhatatbeh A. et al., 2026). Phenotypes may also serve as enrichment or stratification factors in pragmatic trials of phenotype-informed care. Integrating these clinical phenotypes with advanced data-driven algorithms, including neural networks and radiomic models, could further enhance early risk stratification and improve the precision of KOA incidence prediction (Chen et al., 2025).

### 4.5 Strengths and limitations

This study has strengths and limitations. Strengths include the large, racially diverse cohort; linkage of deep learning–derived Kellgren–Lawrence grades with longitudinal EHR data; comorbidity definitions from pre-index diagnoses; and highly stable clusters on repeated random 80% subsampling. Limitations include the single academic health system, retrospective design, and reliance on diagnosis codes and problem lists, which may misclassify conditions such as obesity, smoking, and depression. The dataset also lacked psychosocial and social determinants of health measures and more advanced imaging data: no MRI metadata/files, segmentation outputs, radiomic features, or compartment-specific structural severity measures were available; more advanced MRI analytics, including radiomics and interpretable machine learning, have shown promise in related musculoskeletal contexts and could complement future phenotype refinement (Alkhatatbeh et al., 2025; Alkhatatbeh et al., 2024). Clustering was cross-sectional at the index radiograph, and phenotype transitions were not modeled over time. Although repeated observations were available for many patients (12,280 had ≥2 frontal radiograph dates with usable KL grades, 20,212 had ≥2 knee-specific pain dates, and 10,506 had both), robust transition analysis would require time-updated feature construction and longitudinal phenotype validation beyond the scope of this revision (Supplementary Table S10). The primary time-to-event analysis used last-observed-contact censoring capped at the CPT ascertainment horizon rather than verified continuous enrollment, which may misclassify follow-up time for some individuals; however, excluding zero-day censored observations did not materially change the results (Supplementary Table S9). Requiring a knee-specific pain score within ±90 days of the index radiograph may also have introduced selection bias, although no additional clustering-feature exclusions occurred after the pain-window restriction and a narrower ±30-day sensitivity analysis yielded similar phenotype structure. The Unknown race category is unlikely to represent benign random missingness and may instead reflect heterogeneous documentation processes and differential follow-up: compared with known-race individuals, patients with Unknown race had substantially shorter observed follow-up and lower incident strict TKA, and the MRKR files available for this study do not document how race was originally collected. Accordingly, this category may not represent a biologically or socially coherent group; race estimates should therefore be interpreted cautiously, and known-race-only sensitivity analyses were used to assess robustness. Finally, important structural determinants of arthroplasty use, including payer/insurance, ZIP/postal or other geographic information, provider/facility identifiers, education, income, neighborhood deprivation, patient preferences, trust in the medical system, and other clinician- or system-level factors, were not available. Thus, the race-related findings should be interpreted as associations rather than causal effects. Nonetheless, the identification of robust, clinically interpretable phenotypes that differ by race and are associated with TKA use suggests that routinely collected EHR and imaging data may help study and monitor disparities in knee OA care and inform future equity-focused interventions.

## 5 Conclusion

Using routine EHR and imaging data, we identified three clinically interpretable knee OA phenotypes that differ in age, structural severity, comorbidities, race, and arthroplasty utilization. The strong association between phenotype membership and time to TKA, independent of KL grade, suggests that these clusters capture meaningful differences in disease expression and care delivery. These findings provide a data-driven framework for studying racial disparities and for developing phenotype-aware OA management strategies that warrant external validation.

## Data Availability

Publicly available datasets were analyzed in this study. This data can be found here: https://registry.opendata.aws/mrkr Nightingale Open Science–Emory Knee Radiograph (MRKR) dataset (DOI: 10.48815/N53W2J). The data was accessed on 05 October 2025.
