# Supplementary material for: Racial disparities in pain and total knee arthroplasty across knee osteoarthritis phenotypes
Source: Front Aging. 2026 May 20;7:1819274. doi: 10.3389/fragi.2026.1819274 (PMC13229871; doi:10.3389/fragi.2026.1819274)
Supplement: Supplementary file 1 [file Table1.pdf]

Supplementary Tables

Racial Disparities in Pain and Total Knee Arthroplasty Across Knee Osteoarthritis Phenotypes

Ahmad Alkhatatbeh; Tariq Alkhatatbeh; Jiechen Chen; Hongjiang Chen; Jiankun Xu and Jun Hu

Contents

1 Supplementary Table S1 2
2 Supplementary Table S2 3
3 Supplementary Table S3 4
4 Supplementary Table S4 5
5 Supplementary Table S5 5
6 Supplementary Table S6 6
7 Supplementary Table S7 7
8 Supplementary Table S8 8
9 Supplementary Table S9 9
10 Supplementary Table S10 10

1 Supplementary Table S1. Comparison of individuals included and excluded according to availability of a knee-specific pain score within ±90 days of the index radiograph

Characteristic	Included with knee-specific pain within ±90 days (n = 20,030)	Excluded from pain subset (n = 13,523)	Standardized mean difference
**Continuous variables**			
Age at index, years	61.95 ± 11.03	60.04 ± 10.73	0.176
Maximum KL grade	2.38 ± 1.09	2.28 ± 1.10	0.084
**Binary variables**			
Incident post-index strict TKA, %	17.3%	14.1%	0.088
Pre-index strict TKA, %	0.0%	0.0%	0.000
Diabetes, %	19.5%	14.3%	0.138
Hypertension (HTN), %	53.3%	44.5%	0.176
Obesity, %	32.5%	23.0%	0.214
Chronic kidney disease (CKD), %	8.9%	5.7%	0.122
Cardiovascular disease (CVD), %	18.5%	15.2%	0.086
Autoimmune disease, %	8.2%	6.5%	0.064
Lower extremity trauma, %	32.7%	30.9%	0.040
Nicotine use (ever), %	16.5%	10.7%	0.172
**Sex, n (%)**			
Female	12,840 (64.1%)	8,284 (61.3%)	
Male	7,190 (35.9%)	5,239 (38.7%)	
**Race, n (%)**			
Black	7,733 (38.6%)	4,475 (33.1%)	
American Indian or Alaska Native	62 (0.3%)	38 (0.3%)	
Asian	760 (3.8%)	441 (3.3%)	
White	9,150 (45.7%)	6,918 (51.2%)	
Multiple	114 (0.6%)	56 (0.4%)	
Native Hawaiian or Other Pacific Islander	33 (0.2%)	29 (0.2%)	
Unknown	2,178 (10.9%)	1,566 (11.6%)	
**Ethnicity, n (%)**			
Hispanic or Latino	563 (2.8%)	297 (2.2%)	
Non-Hispanic or Latino	15,934 (79.6%)	10,562 (78.1%)	
Unknown	3,533 (17.6%)	2,664 (19.7%)	

Continuous variables are presented as mean ± standard deviation (SD), and categorical variables are presented as counts and percentages. Standardized mean differences are shown for continuous and binary summary variables. Included individuals had at least one knee-specific pain score within ±90 days of the index radiograph; excluded individuals did not. Comparisons are provided to assess potential selection bias related to pain-score availability.
Abbreviations: KL grade, Kellgren–Lawrence grade; TKA, total knee arthroplasty.

2 Supplementary Table S2. Missingness of clustering features in the full cohort and ±90-day pain subset

Clustering feature	Full cohort missing, n (%)	±90-day pain subset missing, n (%)
Age at index	0 (0.0%)	0 (0.0%)
Maximum Kellgren–Lawrence grade	0 (0.0%)	0 (0.0%)
Median knee pain score	13,523 (40.3%)	0 (0.0%)
Proportion of knee pain scores ≥7	13,523 (40.3%)	0 (0.0%)
Diabetes	0 (0.0%)	0 (0.0%)
Hypertension (HTN)	0 (0.0%)	0 (0.0%)
Obesity	0 (0.0%)	0 (0.0%)
Chronic kidney disease (CKD)	0 (0.0%)	0 (0.0%)
Cardiovascular disease (CVD)	0 (0.0%)	0 (0.0%)
Autoimmune disease	0 (0.0%)	0 (0.0%)
Lower extremity trauma	0 (0.0%)	0 (0.0%)
Nicotine use (ever)	0 (0.0%)	0 (0.0%)

The table summarizes missingness for all features used in the clustering analysis in the full 33,553-individual cohort and in the ±90-day pain subset. Clustering features were age at index, maximum Kellgren–Lawrence grade, median knee pain score, proportion of knee pain scores ⩾7, hypertension, obesity, diabetes, cardiovascular disease, chronic kidney disease, autoimmune disease, lower extremity trauma, and nicotine use. After restriction to individuals with at least one knee-specific pain score within ±90 days of the index radiograph, no additional exclusions occurred because of missing clustering features.

3 Supplementary Table S3. Comparison of individuals with Unknown versus known race in the full MRKR knee OA cohort

Characteristic	Unknown race n = 3,744	Known race n = 29,809
**Continuous variables**		
Age at index, years	58.84 ± 10.56	61.48 ± 10.96
Maximum KL grade at index	2.35 ± 1.05	2.34 ± 1.10
Follow-up time to strict event/censor, days	615.65 ± 825.23	1394.89 ± 1328.92
**Binary variables**		
Pain score within ±90 days, %	2,178/3,744 (58.2%)	17,852/29,809 (59.9%)
Pain score within ±30 days, %	2,019/3,744 (53.9%)	16,063/29,809 (53.9%)
Incident post-index strict TKA, %	256/3,744 (6.8%)	5,127/29,809 (17.2%)
**Comorbidities**		
Hypertension (HTN), %	693/3,744 (18.5%)	16,004/29,809 (53.7%)
Obesity, %	446/3,744 (11.9%)	9,181/29,809 (30.8%)
Diabetes, %	259/3,744 (6.9%)	5,587/29,809 (18.7%)
Cardiovascular disease (CVD), %	140/3,744 (3.7%)	5,618/29,809 (18.8%)
Chronic kidney disease (CKD), %	61/3,744 (1.6%)	2,492/29,809 (8.4%)
Autoimmune disease, %	91/3,744 (2.4%)	2,433/29,809 (8.2%)
Lower extremity trauma, %	987/3,744 (26.4%)	9,749/29,809 (32.7%)
Nicotine use (ever), %	167/3,744 (4.5%)	4,585/29,809 (15.4%)
**Sex, n (%)**		
Female	2,099/3,744 (56.1%)	19,025/29,809 (63.8%)
Male	1,645/3,744 (43.9%)	10,784/29,809 (36.2%)
Ethnicity, n (%)		
Hispanic or Latino	264/3,744 (7.1%)	596/29,809 (2.0%)
Non-Hispanic or Latino	517/3,744 (13.8%)	25,979/29,809 (87.2%)
Unknown	2,963/3,744 (79.1%)	3,234/29,809 (10.8%)
**Primary phenotype among individuals with nonmissing phenotype assignment, n (%)**		
Structural/metabolic advanced OA (phenotype 0)	508/2,178 (23.3%)	6,150/17,852 (34.4%)
Younger, trauma-associated mild OA (phenotype 1)	865/2,178 (39.7%)	5,269/17,852 (29.5%)
Pain-dominant OA (phenotype 2)	805/2,178 (37.0%)	6,433/17,852 (36.0%)

Continuous variables are presented as mean ± standard deviation (SD), and categorical variables are presented as counts and percentages. Known race includes all non-Unknown race categories recorded in MRKR demographics files. Phenotype rows are restricted to individuals with a nonmissing primary phenotype assignment, corresponding to the ±90-day pain subset. Strict TKA indicates CPT-defined total knee arthroplasty using codes 27447, 27486, and 27487. Abbreviations: KL, Kellgren–Lawrence; TKA, total knee arthroplasty; OA, osteoarthritis.

4 Supplementary Table S4. Proportional hazards diagnostics for the primary Cox model of incident post-index strict total knee arthroplasty

Variable	Pearson correlation with log (time)	P value
Maximum KL grade (per SD)	-0.0005	0.990
Median knee pain (per SD)	0.0415	0.301
Age at index (per SD)	-0.0121	0.762
Younger, trauma-associated mild OA (phenotype 1)	-0.0173	0.662
Pain-dominant OA (phenotype 2)	0.0024	0.953
Male (ref: Female)	-0.0271	0.503
American Indian or Alaska Native (ref: Black)	-0.0040	0.920
Asian (ref: Black)	-0.0018	0.964
White (ref: Black)	-0.0151	0.709
Multiple (ref: Black)	0.0351	0.384
Native Hawaiian or Other Pacific Islander (ref: Black)	-0.0377	0.348
Unknown (ref: Black)	-0.0456	0.254

Diagnostics are from the primary multivariable Cox proportional hazards model for incident post-index strict TKA among individuals in the pain subset without pre-index strict TKA. The table summarizes proportional hazards assessment for each covariate using the correlation between scaled Schoenfeld residuals and log(time). No covariate showed evidence of violation of the proportional hazards assumption at $\alpha = 0.05$. TKA indicates total knee arthroplasty.
Abbreviations: KL grade, Kellgren–Lawrence grade; SD, standard deviation; OA, osteoarthritis; TKA, total knee arthroplasty.

5 Supplementary Table S5. Phenotype-specific follow-up and Kaplan–Meier cumulative incidence of incident post-index strict total knee arthroplasty in the primary Cox cohort

Characteristic	Phenotype 0<br>n = 6,657	Phenotype 1<br>n = 6,133	Phenotype 2<br>n = 7,237
Phenotype label	Structural/metabolic advanced OA	Younger, trauma-associated mild OA	Pain-dominant OA
**Outcome summary**			
Incident strict TKA, n (%)	1,819 (27.3%)	429 (7.0%)	1,224 (16.9%)
Censored, n (%)	4,838 (72.7%)	5,704 (93.0%)	6,013 (83.1%)
Median follow-up to strict event/censor, days	784	925	797
**Kaplan–Meier cumulative incidence of incident strict TKA**			
1-year cumulative incidence, %	20.1%	4.3%	12.6%
3-year cumulative incidence, %	27.8%	6.9%	18.3%

Summaries are restricted to the primary Cox cohort, defined as individuals in the ±90-day pain subset without pre-index strict total knee arthroplasty. Incident strict TKA indicates CPT-defined total knee arthroplasty using codes 27447, 27486, and 27487. Cumulative incidence values are unadjusted Kaplan–Meier estimates at 1 and 3 years. Percentages may not sum to exactly 100% because of rounding.
Abbreviations: TKA, total knee arthroplasty; OA, osteoarthritis; CPT, Current Procedural Terminology.

6 Supplementary Table S6. Pain-window timing summary and ±30-day phenotype sensitivity analysis

Characteristic	±30-day window	±90-day window
**Pain-window timing summary**		
Individuals with knee-specific pain data, n	18,082	20,030
Closest pain score recorded on the index date, n	15,556	15,556
Closest pain score before the index date, n	995	1,379
Closest pain score after the index date, n	1,531	3,095
Closest pain score within 7 days of the index date, n	16,515	16,515
Median closest offset from the index date, days	0	0
Median offset of the median pain measure from the index date, days	0	0
90th percentile of the median pain-measure offset, days	11	44

**Clustering sensitivity**	**Value**
Individuals overlapping between ±30-day and ±90-day phenotype solutions, n	18,082
Adjusted Rand index for overlap between ±30-day and ±90-day solutions	0.808
Mapped label agreement between ±30-day and ±90-day solutions	93.4%
Silhouette coefficient, ±30-day solution	0.219
Silhouette coefficient, ±90-day solution	0.211
Calinski-Harabasz index, ±30-day solution	5951.37
Calinski-Harabasz index, ±90-day solution	6438.63

**Phenotype summaries for the ±30-day solution mapped to the primary ±90-day labels**	**n**	**Age at index, years**	**Maximum KL grade**	**Median knee pain (0–10)**
Structural/metabolic advanced OA (phenotype 0)	5,635	70.0	2.82	3.98
Younger, trauma-associated mild OA (phenotype 1)	5,189	54.1	1.68	3.97
Pain-dominant OA (phenotype 2)	7,258	61.4	2.49	8.14

The table summarizes timing of knee-specific pain scores relative to the index radiograph and the sensitivity analysis using a narrower ±30-day pain window. It includes the number of individuals with pain data within ±30 days, the overlap between the ±30-day and ±90-day phenotype solutions, and agreement metrics for phenotype assignment. Where shown, phenotype summaries for the ±30-day solution are provided to assess robustness of the primary ±90-day analysis. Values for phenotype summaries are means unless otherwise indicated.

Abbreviations: KL grade, Kellgren–Lawrence grade; TKA, total knee arthroplasty; OA, osteoarthritis.

7 Supplementary Table S7. Known-race-only Cox proportional hazards model for incident post-index strict total knee arthroplasty

Variable	Hazard ratio (95% CI)	P value
Age at index (per SD)	0.96 (0.92–1.00)	0.071
Maximum KL grade (per SD)	2.00 (1.91–2.09)	<0.001
Median knee pain (per SD)	1.06 (1.00–1.13)	0.040
**Phenotype (ref: structural/metabolic advanced OA; phenotype 0)**		
Younger, trauma-associated mild OA (phenotype 1)	0.49 (0.43–0.56)	<0.001
Pain-dominant OA (phenotype 2)	0.72 (0.64–0.82)	<0.001
**Sex**		
Male (ref: Female)	1.15 (1.07–1.23)	<0.001
**Race (ref: Black)**		
American Indian or Alaska Native	1.55 (0.92–2.63)	0.102
Asian	1.47 (1.24–1.76)	<0.001
White	1.51 (1.40–1.63)	<0.001
Multiple	1.70 (1.09–2.64)	0.019
Native Hawaiian or Other Pacific Islander	1.42 (0.63–3.16)	0.396

Hazard ratios are from a multivariable Cox proportional hazards model restricted to individuals with known race categories. The model includes age at index, maximum KL grade, median knee pain score, phenotype, sex, and race. Follow-up began at the index radiograph date and ended at incident strict TKA or censoring at last observed contact, capped at the CPT ascertainment horizon. Continuous predictors were standardized. Reference categories are indicated in parentheses.

Abbreviations: CI, confidence interval; SD, standard deviation; KL grade, Kellgren–Lawrence grade; TKA, total knee arthroplasty; OA, osteoarthritis; CPT, Current Procedural Terminology.

8 Supplementary Table S8. Cox proportional hazards model for incident post-index strict total knee arthroplasty with categorical maximum Kellgren–Lawrence grade

Variable	Hazard ratio (95% CI)	P value
Age at index (per SD)	0.98 (0.94–1.01)	0.214
Median knee pain (per SD)	1.04 (0.98–1.10)	0.182
**Maximum KL grade (ref: KL grade 2)**		
KL grade 3	2.18 (1.91–2.49)	<0.001
KL grade 4	4.61 (4.05–5.25)	<0.001
**Phenotype (ref: structural/metabolic advanced OA; phenotype 0)**		
Younger, trauma-associated mild OA (phenotype 1)	0.49 (0.43–0.56)	<0.001
Pain-dominant OA (phenotype 2)	0.76 (0.67–0.87)	<0.001
**Sex**		
Male (ref: Female)	1.13 (1.06–1.22)	<0.001
**Race (ref: Black)**		
American Indian or Alaska Native	1.57 (0.92–2.67)	0.100
Asian	1.47 (1.23–1.75)	<0.001
White	1.47 (1.36–1.59)	<0.001
Multiple	1.71 (1.09–2.68)	0.020
Native Hawaiian or Other Pacific Islander	1.39 (0.62–3.12)	0.423
Unknown	1.00 (0.85–1.17)	0.996

Hazard ratios are from a multivariable Cox proportional hazards model in which maximum KL grade was modeled categorically rather than as a standardized continuous term. The model otherwise includes median knee pain score, phenotype, age at index, sex, and race. Follow-up began at the index radiograph date and ended at incident strict TKA or censoring at last observed contact, capped at the CPT ascertainment horizon. Reference categories are indicated in parentheses.

Abbreviations: CI, confidence interval; SD, standard deviation; KL grade, Kellgren–Lawrence grade; TKA, total knee arthroplasty; OA, osteoarthritis; CPT, Current Procedural Terminology.

9 Supplementary Table S9. Sensitivity analysis excluding zero-day censored observations in the primary Cox model of incident post-index strict total knee arthroplasty

Variable	Primary model Hazard ratio (95% CI)	P value	Sensitivity model excluding zero-day censored observations Hazard ratio (95% CI)	P value
Age at index (per SD)	0.974 (0.936–1.014)	0.204	0.974 (0.936–1.014)	0.204
Maximum KL grade (per SD)	2.003 (1.918–2.093)	<0.001	2.011 (1.925–2.102)	<0.001
Median knee pain (per SD)	1.051 (0.993–1.112)	0.083	1.051 (0.993–1.111)	0.083
**Phenotype (ref: structural/metabolic advanced OA; phenotype 0)**				
Younger, trauma-associated mild OA (phenotype 1)	0.474 (0.417–0.539)	<0.001	0.474 (0.417–0.539)	<0.001
Pain-dominant OA (phenotype 2)	0.732 (0.650–0.825)	<0.001	0.732 (0.650–0.825)	<0.001
**Sex**				
Male (ref: Female)	1.143 (1.065–1.227)	<0.001	1.143 (1.065–1.227)	<0.001
**Race (ref: Black)**				
American Indian or Alaska Native	1.548 (0.914–2.623)	0.104	1.548 (0.914–2.623)	0.104
Asian	1.475 (1.235–1.761)	<0.001	1.475 (1.235–1.761)	<0.001
White	1.501 (1.389–1.621)	<0.001	1.501 (1.389–1.621)	<0.001
Multiple	1.703 (1.094–2.650)	0.018	1.703 (1.094–2.650)	0.018
Native Hawaiian or Other Pacific Islander	1.415 (0.634–3.158)	0.396	1.415 (0.634–3.158)	0.396
Unknown	0.984 (0.843–1.149)	0.839	0.984 (0.843–1.149)	0.840

Hazard ratios are from multivariable Cox proportional hazards regression models for incident post-index strict TKA adjusted for age at index, maximum KL grade, median knee pain, phenotype, sex, and race. The primary model included 20,027 individuals with 3,472 incident strict-TKA events. The sensitivity model excluded 1,070 individuals censored on the index date, leaving 18,957 individuals and the same 3,472 incident events. Excluding zero-day censored observations did not materially change the primary estimates.
Abbreviations: CI, confidence interval; SD, standard deviation; KL grade, Kellgren–Lawrence grade; TKA, total knee arthroplasty; OA, osteoarthritis.

10 Supplementary Table S10. Availability of repeated longitudinal observations in the final MRKR knee OA cohort (N = 33,553)

Characteristic	Value
Patients with ≥2 unique frontal radiograph dates with nonmissing KL, n (%)	12,280 (36.6%)
Patients with ≥2 unique knee-pain dates, n (%)	20,212 (60.2%)
Patients with both ≥2 radiograph dates and ≥2 knee-pain dates, n (%)	10,506 (31.3%)
Patients with ≥2 unique ICD dates, n (%)	33,072 (98.6%)
Patients with ≥2 unique CPT dates, n (%)	32,261 (96.1%)

The table summarizes patient-level availability of repeated dated observations in the final MRKR knee OA cohort. Repeated radiograph dates were defined using frontal radiograph records with nonmissing KL grade in MRKR image metadata. Repeated knee-pain dates were defined using knee-specific pain records in MRKR pain tables. Repeated diagnosis and procedure dates were defined using dated rows in the MRKR ICD and CPT tables, respectively. Percentages are calculated using the full final cohort (N = 33,553) as the denominator.

Abbreviations: MRKR, the Emory Knee Radiograph dataset; OA, osteoarthritis; KL grade, Kellgren–Lawrence grade; ICD, International Classification of Diseases; CPT, Current Procedural Terminology
