# Supplementary figures and images for: Racial disparities in pain and total knee arthroplasty across knee osteoarthritis phenotypes
Source: Front Aging. 2026 May 20;7:1819274. doi: 10.3389/fragi.2026.1819274 (PMC13229871; doi:10.3389/fragi.2026.1819274)

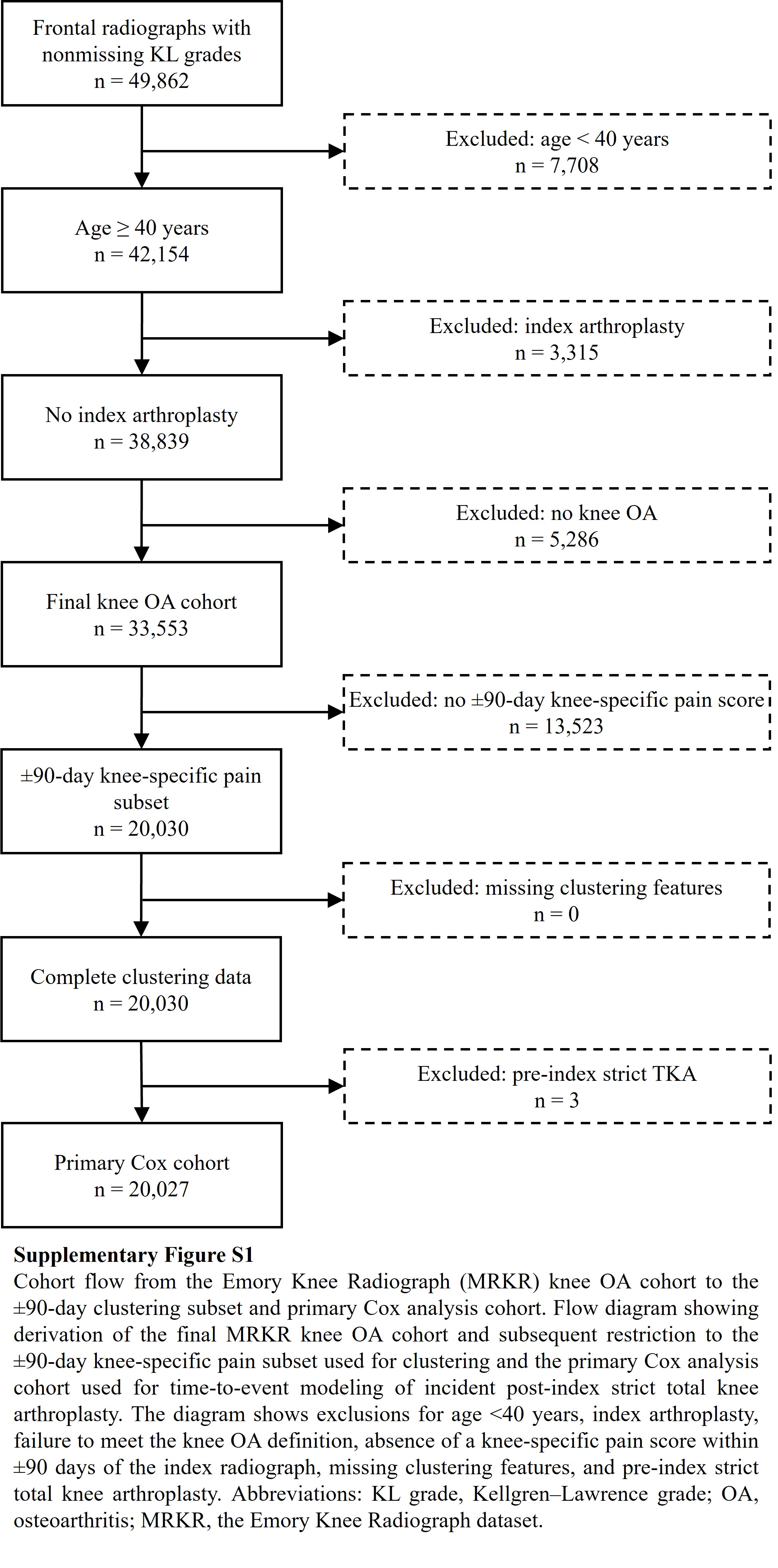

Supplement: Supplementary file 2 [file Image1.jpg]
